# Combining Ability and Performance of Extra-Early Maturing Provitamin A Maize Inbreds and Derived Hybrids in Multiple Environments

**DOI:** 10.3390/plants11070964

**Published:** 2022-04-01

**Authors:** Olatise Oluwaseun, Baffour Badu-Apraku, Moses Adebayo, Adamu Masari Abubakar

**Affiliations:** 1 Department of Crop Production and Soil Science, Ladoke Akintola University of Technology, Ogbomoso PMB 4000, Nigeria; olatiselola@gmail.com (O.O.); moses.adebayo@seedcogroup.com (M.A.); 2 International Institute of Tropical Agriculture, Oyo Road, Ibadan PMB 5320, Nigeria; a.abubakar@cgiar.org

**Keywords:** *Zea mays* L., line × tester design, provitamin A

## Abstract

Availability of maize (*Zea mays* L.) hybrids with elevated provitamin A (PVA) levels and tolerance to contrasting stresses would improve food self-sufficiency and combat malnutrition in sub-Saharan Africa (SSA). This study was conducted to (i) analyze selected PVA inbreds of extra-early maturity for carotenoid content, (ii) estimate the combining abilities of the inbred lines for grain yield and other agronomic traits, (iii) assign inbred lines to distinct heterotic groups (HGs), (iv) identify testers among the inbred lines, and (v) determine grain yield and stability of the PVA hybrids across contrasting environments. Thirty-three extra-early maturing inbred lines selected for high carotenoid content were crossed with four inbred testers to obtain 132 testcrosses. The testcrosses, six tester × tester crosses and two hybrid checks, were evaluated across three *Striga*-infested, four drought and five optimal growing environments in Nigeria, 2014–2016. Results of the chemical analysis revealed that inbred lines TZEEIOR 109, TZEEIOR 30, TZEEIOR 41, TZEEIOR 97, TZEEIOR 42, and TZEEIOR 140 had intermediate PVA levels. Both additive and nonadditive gene actions were important in the inheritance of grain yield and other measured traits under stress and optimal environments. However, additive gene action was preponderant over the nonadditive gene action. The inbred lines were classified into three HGs across environments. Inbreds TZEEIOR 249 and TZEEIOR 30 were identified as testers for HGs I and II, respectively. The hybrid TZEEI 79 × TZEEIOR 30 was the most outstanding in terms of grain yield and was stable across environments. This hybrid should be tested extensively in on-farm trials for consistency in performance and commercialized to combat malnutrition and food insecurity in SSA.

## 1. Introduction

Maize (*Zea mays* L.), an important staple food, feed, and industrial crop, is widely adapted and cultivated in SSA [1]. However, it is deficient in essential micronutrients, particularly provitamin A (PVA), which can be obtained from only external food sources because the human body cannot synthesize it. Vitamin A is involved in various biological processes such as vision, growth, and immunity to diseases. West and Darnton [2] indicated that deficiency of vitamin A (VAD) is responsible for night blindness and may lead to stunted growth in vulnerable children. It can also weaken the immune system, as well as increase mortality resulting from infectious diseases such as malaria, diarrhea, and measles [3]. Maize biofortification with superior amounts of PVA is the most promising option for alleviating the health problems associated with VAD [4]. It has been found that children who consume PVA-enriched maize varieties have total body reserves of vitamin A comparable to what is obtainable through fortification and supplementation [5]. However, according to [6], yellow endosperm cultivars of maize common with farmers worldwide contain 2 μg·g^−1^ of PVA, which is far below the daily requirement in a diet [7]. Consequently, researchers have devoted enormous efforts toward increasing the concentration of PVA carotenoids in maize through traditional plant breeding to combat VAD [5].

The West and Central Africa (WCA) savannas have high incoming solar radiation and low pest and disease incidence, which lead to high crop yield. As a result, the savannas can be explored for the attainment of self-sufficiency of food throughout the sub region. However, factors such as *Striga* parasitism and recurrent water deficit limit maize grain yield in the savanna agro-ecologies of SSA. Severe *Striga* infestation can lead to complete crop failure and compel maize farmers to abandon their farmlands. Although there are several methods of *Striga* control [8], but improving the resistance and tolerance of host plants to the parasitic plant is the economical and most sustainable strategy for alleviating the adverse effects of the weed on maize [9]. Therefore, maize hybrids and varieties resistant to *Striga* have been used as an integrated *Striga* control strategy in SSA [10]. Thus, the most economically feasible and sustainable *Striga* control method is the host plant resistance approach [11,12]. DeVries [11] reported drought as another major cause of yield instability in SSA that can lead to about 90% reduction in maize grain yield. Similarly, Badu-Apraku et al. [9] reported 42% reduction in maize yield under *Striga* parasitism and 53% reduction under moisture stress. *Striga* parasitism often occurs simultaneously with water deficit under field conditions and reduces grain yield dramatically [10]. Although several early (90–95 days to physiological maturity) and extra-early (80–85 days to physiological maturity) white and yellow endosperm varieties and hybrids of maize with improved *Striga* resistance/tolerance and drought tolerance have been developed and commercialized in SSA [13]. However, no maize hybrid with *Striga* resistance/tolerance, drought tolerance, extra-earliness, and high provitamin A is available. Such hybrids, if developed and commercialized in SSA, will help to simultaneously address devastating effects of *Striga* and drought stresses, and mitigate the adverse effects of PVA deficiency in SSA. Thus, the development and deployment of germplasms with enhanced *Striga* resistance and tolerance to suboptimal water levels are crucial for sustainable maize production and productivity. Therefore, the ideal maize variety or hybrid for commercialization in SSA should have enhanced levels of PVA and tolerance/resistance to *Striga*, as well as tolerance to drought.

The combining ability of inbred lines of maize and the classification of inbred lines into HGs provide information on their usefulness for the development of productive hybrids. The potentials of inbred lines to hybridize in such a way that favorable genes/characters are passed on to their progenies is referred to as combining ability [14]. Studies on combining abilities are invaluable for designing hybrid breeding programs and comparing the performance of hybrids derived from the inbred lines. An assessment of the general combining ability (GCA) effects of inbred lines for grain yield and other agronomic traits relative to the specific combining ability (SCA) effects of the derived hybrids is crucial for the identification of outstanding hybrids for commercialization [15]. Information on the combining ability of inbred lines in hybrid combinations under the prevailing environmental conditions is, therefore, necessary for a successful maize hybrid improvement. Different reports on gene action controlling *Striga* resistance/tolerance and drought tolerance for grain yield and other traits of normal endosperm maize inbred lines are very well documented. Additive genetic effects have been reported to be more important than nonadditive effects in controlling *Striga* resistance in normal endosperm extra-early white inbred lines [16,17], whereas nonadditive gene action played a greater role in genetic control of *Striga* resistance indicator traits in extra-early white endosperm maize [18,19]. Contrasting findings have been reported on the genetic effects controlling maize grain yield under drought management conditions. It is, therefore, of utmost importance for breeding programs to study the GCA of inbred lines to be used as parents in hybrid combinations and to obtain information on SCA and heterotic patterns. Consequently, combining ability studies of inbred lines are routinely carried out to identify parental lines that could be used in developing productive hybrids [20]. Such studies are also essential in plant breeding programs for assessing the superiority of parental lines in hybrid combinations [15,21]. Results of combining ability studies under drought stress have indicated the predominance of SCA over GCA effects for grain yield, anthesis–silking interval, days to silking, plant height, plant and ear aspects, root lodging, and ears per plant under drought conditions. For example, nonadditive gene action has been reported to be more important than additive gene action for grain yield under drought management conditions [1,22]. Contrarily, other researchers [23,24,25] have reported the preponderance of additive gene action for grain yield and other traits under drought management conditions. The contrasting results may be attributed to the differences in the sources and genetic background of the inbred lines used for the different studies, the intensity of drought stress conditions, and the influence of environmental conditions, such as soil and climate.

Results of several genetic studies have revealed that GCA is the major component accounting for the differences among diallel crosses or testcrosses under *Striga hermonthica* infestation in the field. Furthermore, high GCA effects in an inbred line under *Striga* infestation are indicative of the performance of the lines and should be a good indicator of the performance of their hybrids, i.e., the probability that the inbred lines will transmit their characteristics to their progenies [1,17]. These results are consistent with those of [19,26,27,28,29]. The authors reported that additive genetic effects were more important in the control of host plant damage syndrome rating and grain yield, whereas nonadditive gene action controlled the number of emerged *Striga* plants. Contrarily, the results of this study disagreed with the findings of [15,27,30], which demonstrated that nonadditive gene action was more important than additive gene action in the control of host plant damage, whereas additive gene action was more important in the control of the number of emerged *Striga* plants. The International Institute of Tropical Agriculture (IITA) Maize Improvement Program (MIP) has developed numerus PVA inbred lines of extra-early maturity, but there are only few reports documented on the combining ability and heterotic patterns of the extra-early maturing PVA inbred lines. The few reports available in the literature on the extra-early PVA inbred lines indicated that information on the combining ability of the IITA-MIP inbred lines is very scarce, and that more information is required for planning crosses among inbreds of opposing heterotic groups for the development of high-yielding PVA hybrids for commercialization in SSA.

The present study was conducted to (i) analyze selected PVA inbreds of extra-early maturity for carotenoid content, (ii) estimate the combining abilities of the inbred lines for grain yield and other agronomic traits, (iii) assign inbred lines to distinct heterotic groups using the GCA effects of multiple traits (HGCAMT) grouping method, (iv) identify testers among the inbred lines, and (v) determine yield and stability of the PVA hybrids across contrasting research environments.

## 2. Results

### 2.1. Chemical Analysis and Response of Inbreds to Stresses

Results of chemical analysis revealed that the PVA contents of the inbred lines varied from 0.86 μg·g^−1^ for TZEEI 63 to 11.57 μg·g^−1^ for TZEEIOR 41 (Table 1). The PVA inbred lines TZEEIOR 41, TZEEIOR 30, TZEEIOR 109, TZEEIOR 42, TZEEIOR 140, and TZEEIOR 97 had PVA contents greater than 10 μg·g^−1^ and were also classified as drought-tolerant and/or *Striga*-tolerant/resistant.

### 2.2. Analysis of Variance for Grain Yield and Other Agronomic Traits

The results of the ANOVA for grain yield and other measured traits across *Striga*-infested environments revealed significant mean squares for environment (E), hybrid (G), and environment × hybrid interaction (GEI) for all measured traits, except for hybrid and GEI mean squares for anthesis silking interval (ASI). Additionally, environment × hybrid interaction (GEI) mean squares were not significant for husk cover (HUSK), ear aspect (EASP), ears per plant (EPP), *Striga* damage rating at 8 weeks after planting (WAP), and emerged *Striga* counts at 8 and 10 weeks after planting (WAP). Similarly, significant mean squares were observed for GCA (line), GCA (tester), and E × GCA (tester) interaction for all measured traits except GCA (line) mean squares for anthesis silking interval (ASI), GCA (tester) mean squares for ear per plant (EPP), and E × GCA (tester) mean squares for days to silking, anthesis silking interval (ASI), husk cover (HUSK), and emerged *Striga* count at 8 weeks after planting and 10 weeks after planting (WAP). Contrarily, the E × GCA (line), and E × SCA (line × tester) mean squares were not significant for the measured traits except E × GCA (line) mean squares for husk cover (HUSK), ear rot, *Striga* damage rating at 8 and 10 weeks after planting (WAP), and E × SCA (line × tester) mean squares for grain yield, husk cover (HUSK), and *Striga* damage rating 10 weeks after planting (WAP). Additionally, SCA (line × tester) mean squares were not significant for measured traits except grain yield (Table 2).

Across drought environments (Table 3), the ANOVA showed significant mean squares for E, G, GEI, GCA (line), GCA (tester), and SCA (line × tester) except for GEI mean squares for grain yield, days to anthesis (DA), anthesis silking interval (ASI), and ear rot and GCA (tester) mean squares for grain yield and ears per plant (EPP). Additionally, E × GCA (line) mean squares were significant except for days to anthesis, anthesis silking interval, ear rot, and stay green characteristic. Contradictorily, E × GCA (tester) and E × SCA (line × tester) mean squares were not significant for E × GCA (tester) mean squares for days to silking (DS), husk cover (HUSK), plant aspect (PASP), ear aspect (ASP), and stay green characteristic (STGR). Similarly, E × SCA (line × tester) mean squares were not significant except for HUSK.

The results of the ANOVA across optimum environments (Table 4) revealed highly significant mean squares for E, G, GCA (line), GCA (tester), and SCA (line × tester) for measured traits except for GCA (line), GCA (tester), and SCA (line × tester) mean squares for anthesis silking interval (ASI) and SCA (line × tester) mean squares for husk cover (HUSK) and ears per plant (EPP). Similarly, significant mean squares were observed GEI, E × GCA (line), and E × GCA (tester) for measured traits except for GEI mean squares for grain yield and husk cover (HUSK), E × GCA (tester) mean squares for anthesis silking interval (ASI) and husk cover (HUSK), and E × GCA (tester) mean squares for husk cover (HUSK),. Contrarily, E × SCA (line × tester) mean square interactions were not significant except for anthesis silking interval (ASI).

### 2.3. GCA Effects of PVA Inbreds under Contrasting Environments

Significant positive GCA effects for grain yield were observed for inbreds TZEEIOR 30, TZEEIOR 97, TZEEIOR 99, TZEEIOR 109, TZEEIOR 139, TZEEIOR 197, TZEEIOR 249, and TZdEEI 7 under optimal environments, TZEEI 58 and TZEEI 79 under induced drought stress, and TZEEIOR 30, TZEEIOR 41, TZEEIOR 140, TZEEIOR 197, TZEEIOR 249, and TZEEIOR 251 under *Striga*-infested environment. Contrarily, significant negative GCA effects for grain yield were recorded for the inbreds TZEEIOR 47, TZEEIOR 125, TZdEEI 9, TZdEEI 13, TZEEI 58, TZEEI 63, TZEEI 68, TZEEI 69, TZEEI 96, and TZEEI 95 under optimal environments, TZEEI 69 and TZEEI 95 under drought stress, and TZEEIOR 123, TZEEIOR 125, TZdEEI 13 TZEEI 69, and TZEEI 95 under *Striga*-infested environment. Several inbred lines had significant negative GCA effects for plant aspect (PASP), including TZEEIOR 125, TZEEIOR 139, TZEEIOR 161, and TZEEI 79 under drought stress, and TZEEIOR 42, TZEEIOR 76, TZEEIOR 140, TZEEIOR 146, TZEEIOR 161, and TZEEI 79 under optimal management conditions (Table 5). Similarly, ear aspect (EASP) showed significant negative GCA effects for TZEEIOR 30, TZEEI 79, TZEEIOR 123, and TZEEIOR 41 under drought stress, and TZEEIOR 30, TZdEEI 7, TZEEIOR 97, TZEEIOR 42, TZEEIOR 140, TZEEIOR 146, TZEEIOR 109, TZEEIOR 197, and TZEEIOR 249 under optimal growing environments. Significant negative GCA effects for the stay green characteristic (STGR) were detected for inbreds TZEEIOR 30, TZEEIOR 123, and TZEEIOR 125 under drought stress. Inbreds TZEEIOR 197, TZEEIOR 41, TZEEI 79, TZEEIOR 140, TZEEIOR 251, TZEEIOR 161, TZEEIOR 42, and TZEEIOR 249 showed significant negative GCA effects for *Striga* damage rating at 8 WAP (SDR1), while TZEEIOR 41, TZEEIOR 140, TZEEIOR 249, TZEEI 79, TZEEIOR 251, and TZEEIOR 197 had significant negative GCA effects for *Striga* damage rating at 10 WAP (SDR2). Additionally, inbreds TZEEIOR 140, TZEEIOR 97, TZEEIOR 109, TZEEI 79, TZEEIOR 197, and TZEEIOR 146 showed significant negative GCA effects for emerged *Striga* plants at 8 WAP (ESP1), while inbreds TZEEIOR 35, TZEEIOR 109, TZEEIOR 140, and TZEEI 79 had significant negative GCA effects for emerged *Striga* plants at 10 WAP (ESP2).

### 2.4. Heterotic Grouping of Inbreds Using the HGCAMT Method

The 33 PVA inbreds and four testers were classified into three HGs across test environments using the HGCAMT grouping method at a 0.35 level of dissimilarity (Table 6). The first group comprised TZdEEI 7, TZEEIOR 11, TZdEEI 12, TZEEIOR 92, TZEEIOR 99, TZEEIOR 102, TZEEIOR 123, TZEEIOR 125, TZEEIOR 41, TZEEI 58, TZEEIOR 161, TZEEIOR 249, and TZEEIOR 251, and the second group consisted of TZEEIOR 30, TZEEIOR 35, TZEEIOR 42, TZEEIOR 47, TZEEIOR 76, TZEEI 79, TZEEI 81, TZEEIOR 97, TZEEIOR 109, TZEEIOR 139, TZEEIOR 140, TZEEIOR 146, and TZEEIOR 197, while TZdEEI 9, TZdEEI 13, TZEEI 69, TZEEI 82, TZEEI 96, TZEEI 63, TZEEI 64, TZEEI 68, TZEEI 73, TZEEI 76, and TZEEI 95 constituted the third group.

#### Identification of Inbred Testers

Using the criteria established by Pswarayi and Vivek and Singh and Chaudhary 1985 [31,32] for the identification of inbred testers, PVA inbred lines TZEEIOR 30 and TZEEIOR 249 were identified as testers for HGs I and II across test environments, respectively. However, no inbred in HG group III satisfied the criteria for selection of a tester (Table 6).

### 2.5. The Proportionate Contribution of GCA (Line), GCA (Tester), and SCA to the Total Sum of Squares

**Figure 1:**
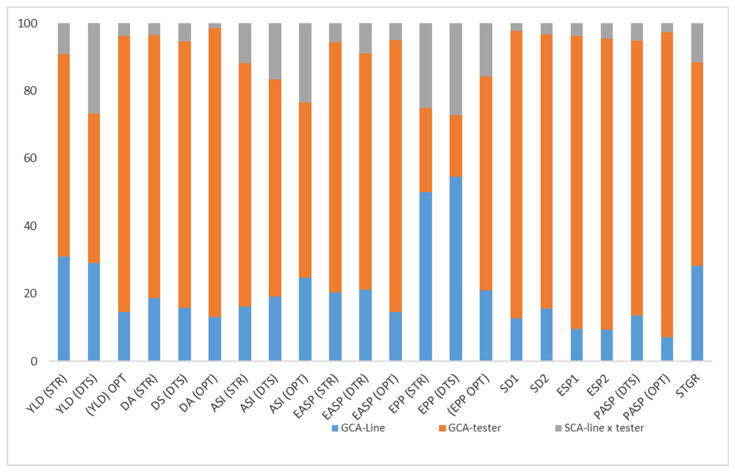
The proportion of total genotypic sum of squares attributable to GCA (line) (lower bar), GCA (tester) (middle bar), and SCA (line × tester) (upper bar) for grain yield and other agronomic of extra-early provitamin A maize inbred lines and four testers. STR: *Striga*-infested, DTS: drought stress, OPT: optimum environment.

Under contrasting environments, the proportion of the GCA sum of squares was greater than the SCA sum of squares for grain yield and other measured traits (Figure 1). GCA accounted for 88% of the total variance for grain yield, 91% for days to 50% anthesis (DA) and days to 50% silking (DS), and 78% and 90% for anthesis silking interval (ASI) and plant height (PLHT), respectively. Partitioning of the total variances into the components, GCA (line), GCA (tester), and SCA (line × tester) revealed that the percentage contributions of the testers were highest for the measured traits in all the test environments except for ear per plant (EPP) under *Striga* infestation and drought stress. The percentage contributions of the testers ranged from 44% for grain yield under drought stress to 90% for plant aspect (PASP) under the optimum environment. The percentage contribution of lines to the total genetic variance ranged from 7% for plant aspect (PASP) under the optimum environment to 55% for ears per plant (EPP) under drought stress. Contrarily, the percentage contributions of the SCA (line × tester) to the total genetic variances were lowest, varying from 2% for days to 50% anthesis (DA) under the optimum environment to 27% for grain yield under drought stress (Figure 1)

### 2.6. Yield Stability of PVA Hybrids across Contrasting Environments

**Figure 2:**
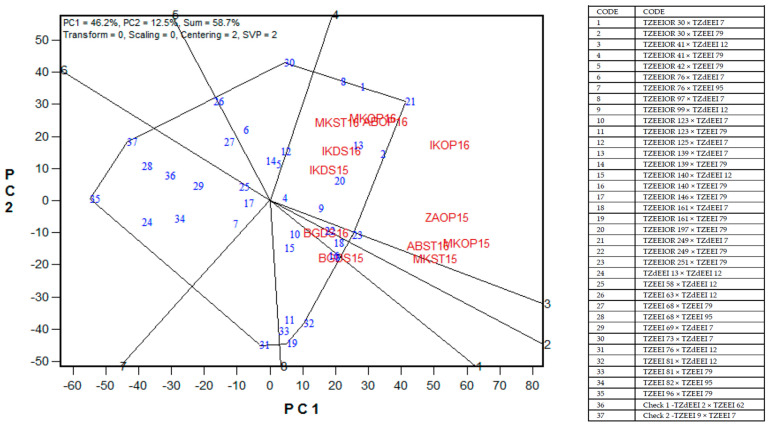
A “which-wins-where or which-is-best-at-what” genotype plus genotype by environment biplot of 35 selected extra-early maturing PVA maize hybrids plus two hybrid checks across stress and non-stress conditions in Nigeria, 2014–2016. ABOP16 = Abuja optimal, 2016; ABST16 = Abuja *Scheme 2016*. BGDS15 = Bagauda drought stress, 2015; BGDS16 = Bagauda drought stress, 2016; IKDS15 = Ikenne drought stress, 2015; IKDS16 = Ikenne drought stress, 2016; IKOP16 = Ikenne optimal, 2016; MKOP15 = Mokwa optimal, 2015; MKOP16 = Mokwa optimal, 2016; MKST15 = Mokwa *Striga*-infested, 2015; MKST16 = Mokwa *Striga*-infested, 2016; ZAOP15 = Zaria optimal, 2015.

**Figure 3:**
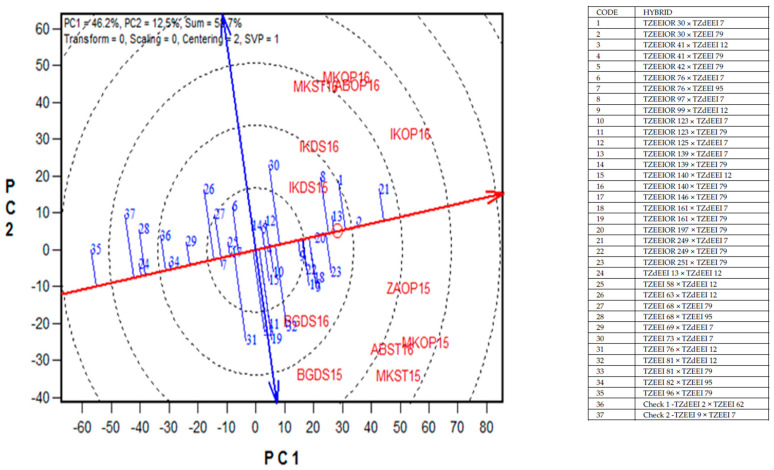
An entry/tester genotype plus genotype × environment interaction biplot of grain yield of 35 selected extra-early maturing PVA maize hybrids and two hybrid checks across stress and non-stress conditions in Nigeria, 2014–2016. ABOP16 = Abuja optimal, 2016; ABST16 = Abuja *Striga*-infested environment, 2016; BGDS15 = Bagauda drought stress, 2015; BGDS16 = Bagauda drought stress, 2016; IKDS15 = Ikenne drought stress, 2015; IKDS16 = Ikenne drought stress, 2016; IKOP16 = Ikenne optimal, 2016; MKOP15 = Mokwa optimal, 2015; MKOP16 = Mokwa optimal, 2016; MKST15 = Mokwa *Striga*-infested, 2015; MKST16 = Mokwa *Striga*-infested, 2016; ZAOP15 = Zaria optimal, 2015.

The polygon view of the GGE biplot for grain yield of 35 (best 20, middle 10, and worst five) selected PVA hybrids with two checks across 12 environments in Nigeria, 2014–2016, is presented in Figure 2. The principal component axes, PC1 and PC2, for Model 3 explained 58.7% of the total variation in grain yield. PVA hybrid 21 (TZEEIOR 249 × TZdEEI 7) was identified as the vertex hybrid for environments IKDS15, IKDS16, MKST16, MKOP16, ABOP16, IKOP16, ZAOP15, MKOP15, ABST16, and MKST15, while PVA hybrid 19 (TZEEIOR 161 × TZEEI 79) was the vertex hybrid for BGDS15 and BGDS16 (Figure 2). Seventeen of the hybrids included in the GGE biplot were not adapted to any of the test environments. In the GGE biplot view, the PVA hybrid 21 (TZEEIOR 249 × TZdEEI 7) had the highest projection on the abscissa and was, therefore, the highest-yielding hybrid across environments (Figure 3). Other top-performing hybrids in terms of grain yield were hybrids 2 (TZEEIOR 30 × TZEEI 79), 1 (TZdEEI 7 × TZEEIOR 30), 13 (TZdEEI 7 × TZEEIOR 139), and 8 (TZEEIOR 97 × TZdEEI 7). Out of the top six high-yielding PVA hybrids, TZEEIOR 30 × TZEEI 79 had the shortest projection from the abscissa and was, therefore, the most stable across contrasting environments. Hybrid 13 (TZEEIOR 139 × TZdEEI 7) was moderately stable and the closest to the ideal hybrid across environments. The GGE biplot view also indicated that more than 80% of the PVA hybrids involved in the GGE biplot analysis out-yielded the two hybrid checks, (TZdEEI 2 × TZEEI 62 and TZEEI 9 × TZEEI 7) across environments.

## 3. Discussion

The presence of drought tolerance and *Striga* resistance/tolerance genes in PVA inbred lines TZEEIOR 97, TZEEIOR 41, TZEEIOR 30, TZEEIOR 140, TZEEIOR 109, and TZEEIOR 42, as well as elevated PVA levels (≥10 μg·g^−1^), indicated that the inbred lines could serve as invaluable sources of genes for developing stress-tolerant hybrids with superior levels of PVA. Furthermore, the PVA inbred lines could be reliable sources of beneficial alleles for the improvement of tropical maize germplasm for *Striga* resistance and elevated levels of PVA for introgression into tropical breeding populations.

The highly significant genotypic mean squares observed for grain yield and other measured traits of the hybrids in the three test environments implied that there was adequate genetic dissimilarity among them to facilitate selection [33]. The significant differences observed among the mean squares for environments for most of the measured traits suggested that the environments were unique and effective in discriminating among the hybrids, thus justifying the need for testing hybrids in diverse environments before making valid conclusions [1]. The significant genotype × environment interaction effects for grain yield and other measured traits under optimal management conditions and *Striga*-infested environments revealed that the hybrids responded differently in varied environments. This confirmed the need for testing hybrids extensively across contrasting environments for many years to select outstanding genotypes for commercialization [16]. However, the lack of significant GEI effects for grain yield and other agronomic traits under drought implied that expressions of the traits were consistent in the drought environments. This also meant that the performance of the hybrids was independent of the test environments.

In a hybrid breeding program, the gene action in the target population or inbred line determines the breeding strategy to be adopted. Baker [34] pointed out that the relative importance of additive gene action (GCA) and nonadditive gene action (SCA) for grain yield indicated the type of gene action in diallel crosses. In the present study, GCA effects of grain yield and other measured traits including *Striga* damage and number of emerged *Striga* plants at 8 and 10 WAP were significantly greater than the SCA effects, suggesting that there was scope for the improvement of most measured traits through selection. This result also implied that there was a chance to identify potentially discriminating testers from non-discriminating testers [35].

The proportion of GCA to SCA for grain yield of the testcrosses across environments was about six times, indicating that the additive gene action was mainly modulating the gene action in the single crosses evaluated in the present study. The preponderance of GCA variances over SCA variances implied that additive gene action was more important than the nonadditive gene action for the measured traits, and that GCA was the major component accounting for the differences among the testcrosses. Additionally, the high GCA variances indicated that the performance of the lines should be a good indicator of the performance of their hybrids, i.e., the inbreds will transmit their characteristics to their progenies [1,17]. These results agree with the findings of [25,26,27,28], which reported that additive genetic variances were more important in the control of host plant damage syndrome rating and grain yield, whereas nonadditive gene action controlled the number of emerged *Striga* plants under *Striga* infestation. On the other hand, the results of the study disagree with the findings of [17,27,30], which demonstrated that nonadditive gene action was more important than additive gene action in the control of the host plant damage, whereas additive gene action was more important in the control of the number of emerged *Striga* plants. The differences in the results of this study and those of earlier workers could be attributed to the fact that the extra-early PVA inbred lines used in the present study and earlier studies were derived from composites of a wide range of germplasms, and these may have had genes with different modes of action from those in inbreds in other studies.

The significant GCA and SCA variances observed for most agronomic traits in *Striga*-infested, managed drought stress, and optimal growing environments indicated that both additive and nonadditive gene actions were modulating the inheritance of the measured traits in the inbreds. However, the predominance of the GCA variances over the SCA for grain yield and other measured traits across test environments suggested that additive gene action was more important than nonadditive gene action. The GCA variances were, therefore, largely responsible for the observed variations among the measured traits of the inbred lines. These results were consistent with earlier reports indicating that inbred lines characterized by high GCA variances contributed significantly to the outstanding grain yield performance of hybrids across contrasting environments [34]. The implication is that population improvement methods such as the S1 family and full-sib selection schemes could be employed to improve the frequency of favorable alleles of heterotic populations derived from the lines in the present study. Another implication of these results is that the testers used were effective in discriminating among the inbred lines in the contrasting environments. Badu-Apraku et al. [29] and Amegbor et al. [16] reported similar findings. However, the results of the present study disagree with those of [36], which reported that nonadditive gene action was more important for grain yield under drought and optimal growing environments. The variations in our results and those of previous researchers may be attributed to the differences in the germplasm used and the contrasting research environments.

The significant interaction of GCA (line or tester) with environment for grain yield and some other measured agronomic traits suggested that the combining abilities of the 33 PVA lines and testers were not consistent in the test environments. However, the lack of a significant interaction of SCA with environment for grain yield and other measured traits across test environments suggested that the discriminating ability of the testers crossed to the inbred lines was not significantly influenced by the contrasting environments.

The GCA effects of grain yield and other measured traits of inbreds provides information on how inbred lines could be used to improve target traits in a population, as well as for developing synthetic varieties and hybrids [16,37]. The significant and positive GCA effects detected for grain yield of inbreds TZEEIOR 249, TZEEIOR 197, TZEEIOR 41, TZEEIOR 30, TZEEIOR 140, and TZEEIOR 251 in *Striga*-infested environments and TZEEI 58 and TZEEI 79 under induced drought stress suggested that these inbred lines possessed beneficial alleles for yield and would be invaluable for developing productive hybrids in *Striga*-infested and drought-prone environments, respectively [35]. Similar results were obtained for the grain yield of the following inbred lines under optimal conditions: TZEEIOR 109, TZdEEI 7, TZEEIOR 30, TZEEIOR 97, TZEEIOR 197, TZEEIOR 99, TZEEIOR 139, and TZEEIOR 249. Of special interest is the inbred TZEEIOR 197, which was identified as possessing genes for high grain yield, drought tolerance, and *Striga* resistance/tolerance, and which was one of the new extra-early PVA inbred testers earlier identified by [38]. This inbred has tremendous potential to contribute to the development of high-yielding, multiple-stress-tolerant hybrids for commercialization in SSA. However, it is striking to note that the two inbred lines, TZEEIOR 97 and TZEEIOR 197, which were identified as inbred testers of opposing heterotic groups by [38], were placed in the same heterotic group with TZEEIOR 30, which was identified as the tester for the HGs II of the present study. There is a need for further studies to confirm this result.

The negative and significant GCA effects obtained for STGR of TZEEIOR 30, TZEEIOR 123, and TZEEIOR 125 under drought environments were an indication of the contribution of these lines to delayed leaf senescence in their progenies. Similarly, GCA effects were detected for SDR1 of inbreds TZEEI 79, TZEEIOR 251, TZEEIOR 41, TZEEIOR 140, TZEEIOR 161, TZEEIOR 197, TZEEIOR 42, and TZEEIOR 249, as well as SDR2 of TZEEI 79, TZEEIOR 140, TZEEIOR 251, TZEEIOR 249, TZEEIOR 41, and TZEEIOR 197. This indicated that the inbreds possessed *Striga* tolerance genes, which could be introgressed into tropical PVA breeding populations for genetic enhancement of *Striga* tolerance and for the development of superior *Striga* tolerant hybrids and synthetic varieties. Additionally, inbreds TZEEIOR 140, TZEEI 79, TZEEIOR 146, and TZEEIOR 109 displayed significant and negative GCA effects for ESP1, while inbreds TZEEIOR 109, TZEEIOR 140, TZEEI 79, and TZEEIOR 35 had significant and negative GCA effects for ESP2, indicating that the inbred lines possessed genes for *Striga* resistance. The inbred tester TZEEI 79 also displayed significant and negative GCA effects for SDR1, SDR2, ESP1, and ESP2, implying that its possessed genes for combined *Striga* resistance and tolerance. The inbred line could, therefore, serve as an invaluable source of beneficial alleles for enhancing combined *Striga* resistance and tolerance in PVA maize breeding populations. These inbred lines offer a great opportunity for improvement of stress tolerance in the extra-early maturing PVA hybrids, which could contribute to improved food self-sufficiency and enhanced nutrition in SSA.

A major objective of this study was to assess the yield performance and stability of the PVA hybrids under contrasting test environments. The PVA hybrid TZEEIOR 161 × TZEEI 79, identified as the most adapted hybrid for two environments (BGDS15 and BGDS16), produced an above average grain yield of the hybrids but was very unstable. Hybrid TZEEIOR30 × TZEEI 79 had outstanding grain yield performance and was the most stable across contrasting environments. The PVA hybrid TZEEIOR 249 × TZdEEI 7 was the most outstanding according to grain yield performance across environments and was moderately stable. Nonetheless, it was close to the ideal hybrid across environments. It was the vertex hybrid in 10 environments and the most outstanding in terms of grain yield performance across environments. This indicated that the PVA hybrids have enormous potential for a significant contribution to increased food and nutritional security in SSA. Therefore, PVA hybrids such as TZEEIOR 249 × TZdEEI 7, TZEEIOR 139 × TZdEEI 7, and TZEEIOR 161 × TZEEI 79 that were high-yielding and stable, as well as adapted to some specific environments, should be further tested in on-farm trials to confirm the consistency of their performance for commercialization in SSA.

## 4. Materials and Methods

### 4.1. Germplasm Development

In 2017, the MIP of IITA established a breeding program that focused on the development of extra-early maize varieties that are multiple-stress-tolerant (i.e., resistant to *Striga* and tolerant to drought and low N) and have high levels of PVA for commercialization in SSA. The *Striga*-resistant extra-early maturing yellow endosperm variety, 2004 TZEE-Y STR C4, was crossed with two high-PVA sources, KU1409/DES1409/(OR2) and Syn–Y-STR-34-1-1-1-1-2-1-B-B-B-B-B/NC354/SYN-Y-STR-34-1-1-1 (OR1), from the MIP so that genes for high carotenoid content could be introgressed into the variety. A generation of backcrossing to the recurrent parent followed to recover extra-earliness. BC1F1 kernels with intense orange color were visually selected and self-pollinated for advancement to the F2 stage and subsequently the F3 stage. The deep-orange colored F3 lines were intercrossed to constitute the extra-early PVA normal endosperm varieties, namely, 2009 TZEE-OR2 STR and 2009 TZEE-OR1 STR. Chemical analysis was not conducted to determine the levels of carotenoids in the PVA varieties due to fund limitations. However, the varieties were tested under *Striga* infestation, drought, and low N starting from 2010, and the results revealed remarkable performance under the contrasting stresses. Additionally, in 2011, an inbred development program was initiated to extract the first set of extra-early maturing maize inbred lines from the high PVA-enriched varieties (2009 TZEE-OR2 STR and 2009 TZEE-OR1 STR). By 2014, S6 in-bred lines had been successfully extracted from the two varieties following repeated self-pollination and visual selection for desirable agronomic characteristics, deep-orange color, as well as shiny yellow to deep-orange kernel endosperm color, and kernel texture varying from semi-flint to completely flint endosperm texture. This was based on the assumption that visual rating for the intense orange kernel endosperm color in maize had fairly high heritability, and that selection for inbred lines with high PVA levels was possible for the deep-orange kernel endosperm color and would lead to elevated levels of the overall amount of carotenoids and increased levels of PVA content [39]. This approach has facilitated rapid and cheap screening of breeding materials in our program and the extraction of PVA inbred lines for hybrid and synthetic variety development. The S6 lines were subsequently evaluated for agronomic performance under contrasting environments. This was followed by the determination of the carotenoid content of the lines using HPLC in the IITA-Ibadan chemical analysis laboratory in 2015. Additionally, the extra-early PVA inbred lines were advanced from the S6–S7 stages through self-pollination during the 2015 growing season to reduce residual heterozygosity. The most promising inbred lines with resistance/tolerance to *Striga*, tolerance to drought and low N, and outstanding levels of carotenoids were selected for the present genetic studies (Table 1).

### 4.2. Generation of Testcrosses

Thirty-three extra-early PVA S7 inbred lines were crossed to four standard extra-early maturing inbred testers of the IITA-MIP, namely, TZEEI 79, TZEEI 95, TZdEEI 7, and TZdEEI 12 (Table 1), in the IITA-Ibadan breeding nursery to obtain 132 testcrosses using the line × tester (L × T) mating design [40].

### 4.3. Production of Kernel Samples for Carotenoid Analysis

Seed samples of testcrosses and inbred lines used for the carotenoid analysis were obtained by selfing the first and last two plants in each plot of the 37 inbred lines (33 extra-early PVA S7 inbred lines plus four inbred testers) and the 132 diallel crosses including the checks as described by [41]. The 33 inbred lines plus the four inbred testers used for diallel crosses, along with the top-performing 13 PVA hybrids, were selected on the basis of the results of genetic studies. Seeds of the inbred lines and hybrids were separately planted under well-watered conditions at Mokwa (9°18′ N, 5°4′ E, altitude 457 m above sea level, 1100 mm annual rainfall) and Ikenne (lat. 6°53′ N, long. 30°42′ E, 60 m above sea level, 1200 mm annual rainfall) in 2016 to produce the kernel samples for the carotenoid analysis of the inbred lines and hybrids according to the procedures described by [41]. Three meter plots with two replications were used for the inbred lines, and 4 m row plots with two replications, each with inter-row and intra-row spacing of 0.75 and 0.40 m, respectively, were used for the inbred and diallel trials at each location. The self-pollinated ears of the inbred lines and hybrids were harvested per plot, dried under ambient temperature, and shelled [42]. Self-pollinated ears of each plot, for each location, were separately harvested, dried, and shelled. The seed samples were stored in the long-term storage facility of IITA at 4 °C. Seed samples obtained from composite seeds that were harvested separately for the inbreds and hybrids were drawn from the long-term storage. Subsequently, the carotenoids were extracted and quantified in the Food and Nutritional Laboratory of IITA, Ibadan, Nigeria. The high-performance liquid chromatography (HPLC) method based on the extraction protocol described by [43] was employed for the carotenoid analysis. The five carotenoids, β-carotene (*cis*- and *trans*-isomers), α-carotene, β-cryptoxanthin, zeaxanthin, and lutein were determined on the basis of calibrations employing external standards. Total carotenoids were computed as the sum of concentrations of α-carotene, β-carotene, lutein, zeaxanthin, and β-cryptoxanthin. PVA levels were computed as the sum of β-carotene and half of each of β-cryptoxanthin and α-carotene contents, because β-cryptoxanthin and α-carotene contribute about 50% of the β-carotene as PVA [44]. Two different measurements were made to represent each sample. The seed samples were processed and stored at 4 °C in the IITA cold store for about 6 months, before random samples of 20–30 maize kernels from the top-yielding and stable PVA hybrids, along with the best yellow endosperm checks obtained from composite grains of the hybrid trials of 2016 at Mokwa and Ikenne, were drawn from the cold room for the analysis of the carotenoids. Only a few hybrid samples were taken for carotenoid analysis due to fund limitations. The IITA laboratory protocol for carotenoid analyses, which involved extraction, separation, and quantification using the HPLC, was employed [45]. The carotenoids that were determined from each sample included lutein, zeaxanthin, beta-carotene (all-*trans*, 9-*cis*, and 13-*cis* isomers) and betacryptoxanthin. The total PVA content of each hybrid was calculated employing the method of [41]. Total PVA = 0.5 (beta-cryptoxanthin) + beta-carotene (all-*trans* + 9-*cis* + 13-*cis* isomers). Total carotenoids were estimated as the sum of concentrations of α-carotene, β-carotene, lutein, zeaxanthin, and β-cryptoxanthin.

### 4.4. Field Experiments

Three field experiments were conducted from 2014 to 2016. The first experiment comprised two trials, one for the inbreds and the other for the hybrids. The inbred trial consisted of 130 extra-early PVA inbred lines (including the four inbred testers and the 33 PVA inbred lines used in the generation of the line × tester crosses). The hybrid trial involved 132 L × T crosses, six tester × tester crosses, and two extra-early yellow normal endosperm commercial hybrid checks. The trials were evaluated for agronomic performance at Bagauda (terminal drought-prone location; latitude 12°00′ N, longitude 8°22′ E, with 580 m altitude and 800 mm yearly rainfall) during the growing seasons of 2015 and 2016. Furthermore, both trials were evaluated at Ikenne (latitude 6°53′ N, longitude 30°42′ E, 60 m above sea level, 1200 mm yearly rainfall) under induced drought stress (DS) during the dry seasons of 2014/2015 and 2015/2016. Drought stress was induced in both trials by irrigating the plants from planting until 21 days after planting (DAP) and suspending irrigation until physiological maturity, so that the plants were dependent on the residual soil moisture for growth and grain filling. A sprinkler irrigation method, which supplied 17 mm of water weekly, from the time the seeds were sown to 3 DAP was used. The soil of the experimental field at Ikenne was characterized as eutric nitrosol [46] with the fields flat, uniform, well-drained, and possessing high water retention capacity. About 60 kg each of N, P, and K per hectare was applied at planting. This was followed by top-dressing of 60 kg of N per hectare at 4 weeks after planting (WAP).

In the second experiment, the inbred lines and hybrids were evaluated separately in adjacent fields artificially infested with *Striga* at Abuja (latitude 9°16′ N, longitude 7°20′ E, 300 m above sea level, 1500 mm yearly rainfall) in 2016 and Mokwa (latitude 9°18′ N, longitude 5°4′ E, 457 m above sea level, 1100 mm yearly rainfall) in 2015 and 2016. Both test locations are in the southern Guinea Savanna of Nigeria. Artificial infestation with *Striga* seeds was carried out according to the procedure described by [47]. The *Striga hermonthica* seeds were obtained from sorghum fields during the previous season and kept for about 6 months before using it for infestation to break the *Striga* seed dormancy. Two weeks before artificial infestation with *Striga*, ethylene gas was applied to the soil to induce suicidal germination of *Striga* seeds existing in the soil to reduce the *Striga* seed bank. Fertilizer application was delayed until about 21–25 DAP before 30 kg·ha^−1^ each of N, P, and K was applied as NPK 15–15–15. Fertilizer application was delayed, and the rate was reduced to stimulate the production of strigolactones and enhance germination of *Striga* seeds, as well as to facilitate the attachment of the emerged *Striga* plants to the roots of the maize plants. Except for *Striga* plants, all other weeds were removed manually.

The third experiment involved evaluation of the PVA hybrids under optimal (OPT) growing environments in Zaria, Abuja, Ikenne, Bagauda, and Mokwa in 2016. The optimal environments involved those sites where moisture was not limiting (well-watered). The hybrid trial at these locations received 60 kg each of N, P, and K per hectare at 3 WAP, and 30 kg of N per hectare was top-dressed at 6 WAP.

In all the three experiments, the inbreds and hybrids were evaluated using 13 × 10 and 14 × 10 lattice designs, respectively, with each replicated twice. The inbred and hybrid trials had 3 and 4 m single-row plots, each with a spacing of 0.75 m and 0.4 m between and within rows. Three seeds were planted per hole, and the seedlings were thinned to two plants per stand, 2 weeks following emergence, to achieve a target population density of about 66,667 plants per hectare. Experiments conducted under drought and optimal growing environments were maintained weed-free through the application of 5 L/ha each of the pre-emergence herbicide Primextra (a.i. atrazine) and the post-emergence herbicide, Gramoxone (a.i paraquat). This was complemented by hand weeding. On the other hand, the *Striga* experiments were kept weed-free using pre-emergence herbicide complemented by hand weeding. Fall armyworms (*Spodoptera frugiperda*) were controlled in all experiments using Ampligo (a.i. 100 g·L^−1^ chlorantraniliprole + 50 g·L^−1^ lamda-cyhalothrin) at 300 mL·ha^−1^.

### 4.5. Data Collection

The procedures and scales used for data collection in the field trials were described in detail by [33]. Briefly, data were recorded for days to 50% silking (DS) and anthesis (DA), anthesis–silking interval (ASI), plant height (PLHT), ear height (EHT), number of ears per plant (EPP), plant aspect (PASP), ear aspect (EASP), husk cover (HUSK), and ear rot (EROT) in all experiments. Moreover, the stay green characteristic (STGR) was scored in drought experiments at 70 DA, while *Striga* damage i.e., SDR1 and SDR2 [38] and number of emerged *Striga* plants, ESP1 and ESP2, were recorded at 8 and 10 WAP in the *Striga* experiments. In the drought-stress experiments, grain yield was estimated from shelled kernel weights per plot, while, under optimal growing and *Striga*-infested environments, grain yield was based on field weights of ears per plot assuming 80% shelling percentage. Grain yield was adjusted to 15% moisture content in all experiments.

### 4.6. Statistical Analysis

Combined analysis of variance (ANOVA) was carried out for agronomic traits of the testcrosses in the *Striga*-infested, managed drought, and optimal growing environments. The logarithmic transformation (*y* + 1) was carried out for count data and arcsine transformation for data in percentages prior to the computation of the ANOVA. The environments and testcrosses were considered as random and fixed effects, respectively, in the combined ANOVA using PROC GLM [48]. Following the estimation of the means obtained from the separate ANOVA for each environment, the line × tester analysis was carried out following the procedure of [31]. The effects of GCA and SCA were computed for grain yield and other measured agronomic traits using the line × tester design.

The method of [34] as modified by [49] was adopted to investigate the importance of the GCA and SCA effects. The HGCAMT grouping method was used to classify the inbreds into heterotic groups across test environments [50]. Ward’s minimum variance cluster analysis based on the Euclidean distance dendrograms generated from HGCAMT was used to place the 33 PVA inbreds plus four testers into heterotic groups across test environments [48]. With the HGCAMT grouping method, the GCA effects of measured traits with significant effects for genotypes across test environments were standardized to obtain a mean of zero and standard deviation of 1. This minimized the effects of different scales of the measured traits. An inbred was classified as a tester if it satisfied the following criteria: (i) displayed significant and positive GCA effects for grain yield, (ii) was classified into a heterotic group, and (iii) had high grain yield [32]. Furthermore, the multiple-trait selection index (MI) involving grain yield, EPP, ASI, PASP, EASP, STGR, SDR1, SDR2, ESP1, and ESP2 under multiple stresses along with grain yield under optimal growing environments was used for characterization of the PVA hybrids for tolerance to multiple stresses [13]. Each trait was standardized to adjust for the effects of the different scales used for measurement of the traits. A positive MI value indicated multiple stress resistance while a negative value indicated susceptibility.

The following equation was used for the computation of the MI:

MI = (2 × YLDSTR) + YLDNSTR + EPP − EASP − PASP − ASI − STGR − SDR1 − SDR2 − (0.5 × ESP1) − (0.5 × ESP2),

where YLDSTR = grain yield across multiple stresses, YLDNSTR = grain yield across optimal growing environments, EPP = number of ears per plant across multiple stresses, ASI = anthesis–silking interval across multiple stresses, EASP = ear aspect across multiple stresses, PASP = plant aspect across multiple stresses, STGR = stay green characteristic across drought and low-N environments, SDR1 and SDR2 = *Striga* damage at 8 and 10 WAP across *Striga*-infested environments, And ESP1 and ESP2 = number of emerged *Striga* plants at 8 and 10 WAP across *Striga*-infested environments.

On the basis of MI, 35 (best 20, middle 10, and worst five) PVA hybrids were selected. The grain yield data of the selected PVA hybrids plus two checks were subjected to GGE biplot analysis to decompose the G × E interactions as described by [51]. The GGE biplot focused on the first two principal components (PC1 and PC2) derived by subjecting the environment centered grain yield means for each location to singular value decomposition. The data were not transformed (transform = 0), were not standardized (scale = 0), and were environment-centered (centering = 2). This provided information on the hybrids that were suitable for the different environments and investigation of stability of hybrids in the various environments.

## 5. Conclusions

Inbred TZEEI 79, an elite yellow inbred tester in the IITA-MIP, showed significant and positive GCA effects for grain yield and significant and negative GCA effects for ESP1, ESP2, SDR1, and SDR2. This indicated that the inbred combined the alleles for *Striga* resistance and tolerance, and that it is an invaluable resource for favorable alleles for genetic enhancement of maize breeding populations in the tropics for resistance/tolerance to *Striga*. The inbreds TZEEIOR 140, TZEEIOR 41, TZEEIOR 197, TZEEIOR 251, and TZEEIOR 249 had significant and positive GCA effects for grain yield under *Striga*, as well as significant and negative GCA effects for SDR1 and SDR2. Contrarily, inbreds TZEEIOR 109 and TZEEIOR 140 displayed significant and negative GCA effects for ESP1 and ESP2. The beneficial alleles from these inbred lines could be introgressed into extra-early maturing tropical PVA breeding populations for genetic enhancement of target traits. The extra-early inbred lines were classified into three HGs with TZEEIOR 249 and TZEEIOR 30 identified as inbred testers for HGs I and II, respectively. The five outstanding PVA hybrids in terms of grain yield, namely, TZEEIOR 30 × TZdEEI 7, TZEEIOR 249 × TZdEEI 7, TZEEIOR 97 × TZdEEI 7, TZEEIOR 139 × TZdEEI 7, and TZEEIOR 30 × TZEEI 79 are recommended for on-farm evaluation to confirm the consistency of their performance in the contrasting environments for commercialization in SSA.

## Figures and Tables

**Table 1:** Description of inbreds used in line × tester study: reaction to stresses and level of provitamin A content.

		Reaction to Stresses		
S/N	Pedigree	Drought	*Striga*	Provitamin-A Content (µg·g^−1^)
1	TZEEIOR 11	S	T	6.48
2	TZEEIOR 30	T	S	10.19
3	TZEEIOR 35	S	S	6.67
4	TZEEIOR 41	S	T	11.57
5	TZEEIOR 42	T	T	10.48
6	TZEEIOR 47	S	S	9.17
7	TZEEIOR 76	S	S	7.77
8	TZEEIOR 92	S	T	7.83
9	TZEEIOR 97	T	R	10.44
10	TZEEIOR 99	T	S	8.77
11	TZEEIOR 102	T	T	4.85
12	TZEEIOR 109	S	T	10.24
13	TZEEIOR 123	T	S	6.25
14	TZEEIOR 125	T	T	4.95
15	TZEEIOR 139	T	T	7.68
16	TZEEIOR 140	T	T	10.32
17	TZEEIOR 146	T	T	7.78
18	TZEEIOR 161	T	S	5.93
19	TZEEIOR 197	S	R	8.45
20	TZEEIOR 249	T	T	6.43
21	TZEEIOR 251	S	T	7.94
22	TZdEEI 9	S	S	4.93
23	TZdEEI 13	T	T	6.29
24	TZEEI 58	T	T	0.97
25	TZEEI 63	T	T	0.86
26	TZEEI 64	T	T	2.45
27	TZEEI 68	T	S	1.63
28	TZEEI 69	T	S	-
29	TZEEI 73	S	T	1.02
30	TZEEI 76	T	S	-
31	TZEEI 81	T	S	1.86
32	TZEEI 82	T	S	1.39
33	TZEEI 96	S	S	1.84
31	TZdEEI 7 (Tester 1)	T	R	6.49
35	TZdEEI 12 (Tester 2)	T	R	5.68
36	TZEEI 79 (Tester 3)	T	T/R	1.12
37	TZEEI 95 (Tester 4)	S	T/R	3.94

S = susceptible, T = tolerant, R = resistant, T/R = resistant and/or tolerant.

**Table 2:** Mean squares from the line × tester analysis of variance of grain yield and other traits of hybrids evaluated across three *Striga*-infested environments in Nigeria during the 2015 and 2016 growing seasons.

Source of Variation	DF	Grain Yield (kg/ha)	Days to Anthesis	Days to Silk	ASI	Husk Cover	Ear Aspect	Ear Rot	Ears/plant	*Striga* Damage		Emerged *Striga* Plants	
										8 WAP	10 WAP	8 WAP	10 WAP
Env (E)	2	8,047,918.6 *	13.28 **	576.47 **	213.61 **	260.59 **	5.73 **	1133.12 **	0.24 **	17.95 **	19.85 **	83.70 **	109.81 **
Rep (Env)	3	11,158,849.6 **	3.59	5.13	2.24	2.24 **	3.46 **	89.10 **	0.010	8.11 **	2.45 **	7.47 **	7.65 **
Block (Rep × Env)	56	5,833,563 **	8.36 **	20.67 **	4.64 **	0.90 **	2.06 **	7.84 **	0.06 **	1.88 **	1.76 **	1.05 **	0.99 **
Hybrid (G)	131	2,653,111.3 **	13.66 **	14.88 **	1.64	0.67 **	1.13 **	4.61 **	0.03 **	1.86 **	1.41 **	0.96 **	0.82 **
GCA (line)	32	6,452,305.8 **	36.35 **	42.56 **	2.41	1.41 **	2.50 **	5.10 *	0.06 **	4.07 **	3.11 **	1.54 **	1.19 **
GCA (tester)	3	12,435,807 **	151.04 **	155.10 **	10.71 **	5.79 **	9.07 **	54.55 **	0.03	27.10 **	16.18 **	14.03 **	10.89 **
E × Hybrid	261	1,254,726.3 **	3.84 **	5.17 **	1.75	0.38	0.52	3.83 **	0.020	0.62	0.62 *	0.56	0.44
E × GCA (line)	64	2,030,161.9	3.99	7.17	1.91	0.80 **	0.65	4.94 *	0.03	1.13 **	1.00 **	0.72	0.58
E × GCA (tester)	6	4,901,911.3 **	13.13 **	11.2	2.55	0.3	1.3	36.71 **	0.10 **	0.65	1.08 **	0.56	0.58
SCA (line × tester)	96	1,905,117.9	6.68 **	8.53	1.76	0.5	0.69	3.74	0.03	0.76	0.66	0.59	0.59
E × SCA (line × tester)	191	1,789,280.5	4.87 **	7.48	1.92	0.55 *	0.79	3.35	0.03	0.87	0.89 **	0.77	0.62
Error	335	951,582	2.81	3.86	4.65	0.33	0.46	2.29	0.020	0.53	0.48	0.52	0.47

*, ** Significant at 0.05 and 0.01 probability levels, respectively; DF = degrees of freedom; Env = environments; Rep = replication.

**Table 3:** Mean squares from the line × tester analysis of variance of grain yield and other traits of 132 hybrids evaluated across drought at four locations in Nigeria during the 2015 and 2016 growing seasons.

Source of Variation	DF	Grain Yield (kg/ha)	Days to Anthesis	Days to Silk	ASI	Husk Cover	Plant Aspect	Ear Aspect	Ear Rot	Ears/Plant	Stay Green Characteristic
Env (E)	3	3,649,642 **	1287.17 **	1940.83 **	76.38 **	1044.14 **	154.50 **	16.97 **	10,124.45 **	6.34 **	194.60 **
Rep (Env)	4	4,357,817	72.23 **	70.48 **	1.92	1.73 **	2.82 **	1.99 **	205.06 **	0.20 **	6.65 **
Block (Rep × Env)	76	3,649,642 **	10.35 **	11.43 **	1.72	0.99 **	0.82 **	1.12 **	15.05 **	0.05 **	2.11 **
Hybrid (G)	131	3,043,437 *	12.06 **	9.27 **	2.31 **	1.26 **	1.27 **	1.06 **	16.98 **	0.06 **	1.70 **
GCA (line)	32	4,271,917 **	25.11 **	19.95 **	2.43 *	2.54 **	2.18 **	2.10 **	27.62 **	0.12 **	3.54 **
GCA (tester)	3	6,453,640	124.39 **	77.96 **	8.12 **	7.00 **	13.01 **	6.87 **	10.99 **	0.04	7.49 **
E × Hybrid	391	2,427,378	2.95	2.74 **	1.16	0.73 **	0.55 **	0.53 *	6.75	0.04 *	0.74
E × GCA (line)	96	4,268,858 **	4.82	6.30 **	1.15	1.31 **	0.96 **	0.93 **	8.64	0.04 *	1.16
E × GCA (tester)	9	1,545,636	7.34	8.10 *	1.93	4.62 **	2.90 **	2.39 **	11.81	0.02	4.91 **
SCA (line × tester)	96	3,912,332 **	8.63 **	7.76 **	2.11 *	1.03 **	0.83 **	0.89 **	16.31 **	0.06 **	1.46 **
E × SCA (line × tester)	286	2,210,299	3.51	3.47	1.18	0.68 *	0.47	0.43	5.66	0.02	0.8
Error	444	2,265,515	2.51	1.91	1.43	0.47	0.38	0.42	7.09	0.02	0.7

*, ** Significant at 0.05 and 0.01 probability levels, respectively; DF = degrees of freedom; Env = environments; Rep = replication.

**Table 4:** Mean squares from the line × tester analysis of variance of grain yield and other traits of 132 hybrids evaluated across optimal conditions at five locations in Nigeria during the 2015 and 2016 growing seasons.

Source of Variation	DF	Grain Yield (kg/ha)	Days to Anthesis	Days to Silk	ASI	Husk Cover	Plant Aspect	Ear Aspect	Ear Rot	Ears/Plant
Env (E)	4	535,147,900 **	749.75 **	1016.97 **	28.02 **	839.50 **	56.23 **	4.94 **	435.76 **	6.37 **
Rep (Env)	5	3,386,843 **	7.27 **	6.50 **	0.19	1.32	4.34 **	0.7	24.48 **	0.07 *
Block (Rep × Env)	94	1,688,063 **	3.70 **	4.19 **	0.31	1.46 *	0.92 **	0.54 **	4.92 **	0.04 *
Hybrid (G)	131	4,317,036 **	9.75 **	10.73 **	0.32	1.47 *	1.31 **	1.41 **	6.87 **	0.04 *
GCA (line)	32	8,837,696 **	23.10 **	25.33 **	0.35	1.35	1.85 **	2.48 **	11.53 **	0.04 *
GCA (tester)	3	49,742,961 **	150.71 **	175.29 **	0.73	10.31 **	23.58 **	13.64 **	67.89 **	0.12 **
E × Hybrid	524	1,112,364	1.59 **	1.84 **	0.35 **	1.17	0.53 **	0.42 *	2.81 **	0.03 **
E × GCA (line)	128	1,558,752 **	2.63 **	3.14 **	0.35	1.18	0.69 **	0.64 **	4.29 **	0.04 *
E × GCA (tester)	12	2,125,208 *	12.87 **	15.83 **	0.71 **	1.67	2.89 **	1.31 **	9.15 **	0.08 *
SCA (line × tester)	96	2,319,543 **	2.72 **	3.18 **	0.33	1.25	0.69 **	0.83 **	3.91 **	0.03
E × SCA (line × tester)	384	1,062,146	1.64	1.87	0.36 **	1.24	0.5	0.42	2.5	0.03
Error	557	915,763	1.08	1.23	0.28	1.09	0.39	0.35	2.07	0.03

*, ** Significant at 0.05 and 0.01 probability levels, respectively; DF = degrees of freedom; Env = environments; Rep = replication.

**Table 5:** General combining ability (GCA) effects of grain yield and other traits of 33 extra-early maturing PVA maize inbred lines and four standard inbred testers evaluated across *Striga*, drought, and optimum conditions.

Line	Grain Yield (kg/ha)			Plant Aspect			Ear Aspect		STGR	*Striga* Damage Rating		Emerged *Striga* Count	
	STR	DT	OPT	STR	DT	OPT	DT	OPT	DT	8 WAP	10 WAP	8 WAP	10 WAP
TZEEIOR 11	131	232	−113	−0.06	−0.13	0.08	−0.14	0.05	−0.14	0.16	0.2	−0.04	−0.03
TZEEIOR 30	616 *	443	635 **	−0.22	−0.24	0.01	−0.40 *	−0.35 **	−0.50 **	−0.22	−0.38	0.06	0.09
TZEEIOR 35	−307	167	206	0.23	0.07	0.09	0.04	−0.18	0	0.2	0.2	−0.24	−0.33 *
TZEEIOR 41	864 **	171	21	−0.47 **	−0.02	−0.16	−0.33 *	−0.05	−0.34	−0.63 **	−0.51 *	0.22	0.23
TZEEIOR 42	−5	144	380	−0.31	−0.09	−0.29 *	−0.27	−0.35 **	−0.28	−0.51 **	−0.3	−0.28	−0.28
TZEEIOR 47	−256	−419	−536 **	−0.06	0.1	−0.01	−0.08	−0.05	0.16	−0.34	−0.09	−0.07	−0.08
TZEEIOR 76	−109	106	−179	−0.06	−0.15	−0.39 **	−0.08	0.02	−0.18	−0.01	−0.05	−0.24	0.07
TZEEIOR 92	253	−81	−236	0.03	0.26	0.29 *	−0.05	0.12	−0.09	0.24	0.07	0.1	0
TZEEIOR 97	468	30	759 **	−0.18	0.01	−0.09	−0.12	−0.28 *	−0.12	−0.38	−0.3	−0.35 *	−0.24
TZEEIOR 99	133	149	580 **	0.23	0.16	−0.21	−0.15	−0.13	−0.28	0.04	−0.01	0.23	0.25
TZEEIOR 102	−331	59	176	0.18	−0.02	0.08	0.07	0.05	0.19	0.48 *	0.36	0.29	0.31 *
TZEEIOR 109	388	125	634 **	−0.31	−0.05	−0.24	−0.15	−0.35 **	0.04	−0.22	−0.26	−0.43 *	−0.44 **
TZEEIOR 123	−779 **	207	−164	0.40 *	−0.27	−0.09	−0.37 *	0.22	−0.64 **	0.54 *	0.49 *	0.05	0.12
TZEEIOR 125	−572 *	218	−384 *	0.44 **	−0.37 *	0.34 **	−0.24	0.30 *	−0.72 **	0.49 *	0.37	0.2	0.04
TZEEIOR 139	−276	236	592 **	0.23	−0.43 *	0.01	−0.12	−0.15	−0.31	−0.01	−0.13	0.02	−0.04
TZEEIOR 140	768 **	−109	232	−0.52 **	−0.24	−0.26 *	−0.08	−0.35 **	0.07	−0.51 *	−0.51 *	−0.51 **	−0.61 **
TZEEIOR 146	−157	166	243	0.07	−0.21	−0.34 **	−0.02	−0.35 **	−0.03	0.16	−0.01	−0.42 *	−0.21
TZEEIOR 161	437	209	243	−0.1	−0.77 **	−0.41 **	−0.12	−0.2	−0.22	−0.51 *	−0.34	0.49 **	0.44 **
TZEEIOR 197	1026 **	−119	770 **	−0.89 **	0.04	−0.11	−0.02	−0.33 **	0.13	−0.63 **	−0.68 **	−0.35 *	−0.16
TZEEIOR 249	758 **	62	717 **	−0.43 **	0.29	−0.11	−0.05	−0.33 **	−0.03	−0.55 *	−0.47 *	0.13	0.26
TZEEIOR 251	682 *	177	256	−0.27	0.2	0.01	0.04	−0.03	−0.06	−0.55 *	−0.55 **	−0.09	−0.05
TZdEEI 9	−343	−190	−456 *	0.15	0.07	0.26 *	0.29	0.32 *	0.1	0.24	0.28	0.29	0.17
TZdEEI 13	−1159 **	−538	−662 **	0.53 **	0.35 *	0.21	0.14	0.2	0.04	1.04 **	0.78 **	0.31	0.2
TZEEI 58	−43	1125 **	−561 **	0.03	−0.3	−0.16	0.07	0.17	0.19	−0.34	−0.3	0.16	0
TZEEI 63	−429	266	−735 **	0.23	−0.09	0.24 **	−0.18	0.47 **	0.07	0.2	0.28	0.01	−0.05
TZEEI 64	−342	−261	−372	−0.1	0.37 *	0.24 **	0.02	0.30 *	0.45 *	0.2	0.32	−0.22	−0.15
TZEEI 68	−386	−249	−497 *	−0.02	0.16	0.11	0.17	0.27 *	0.29	0.45 *	0.32	0.31	0.23
TZEEI 69	−681 *	−1048 **	−821 **	0.40 *	0.48 **	0.39 **	0.82 **	0.27 *	0.79 **	0.37	0.45 *	0.12	0.05
TZEEI 73	−196	−279	−158	0.34 *	0.23	0.04	0.32	0.15	0.69 **	0.31	0.25	0.18	0.05
TZEEI 76	356	84	−159	0.07	0.1	0.19	0.07	0.27 *	0	−0.22	−0.05	0.13	0.14
TZEEI 81	189	−75	201	−0.22	0.13	0.06	−0.08	−0.05	−0.15	−0.13	−0.09	−0.26	0.03
TZEEI 82	−236	−428	−195	0.15	0.13	0.06	0.39 *	0.2	0.22	0.24	0.32	0.24	0.15
TZEEI 96	−501	−550	−431 *	0.53 **	0.23	0.24 **	0.57 **	0.15	0.63 **	0.41	0.37	0.01	−0.1
TZdEEI 7 (T1)	107	103	519 **	−0.04	−0.02	−0.1	−0.06	−0.18 **	−0.15	0	0.01	0.32 **	0.29 **
TZdEEI 12 (T2)	−57	−76	15	0.08	0.02	−0.1	0.14	−0.06	0.17	0.1	0.08	0.05	0.05
TZEEI 79 (T3)	127	156 *	−117	−0.27 **	−0.27 **	−0.20 *	−0.21 *	−0.06	−0.14	−0.50 **	−0.39 **	−0.34 **	−0.29 **
TZEEI 95 (T4)	−176	−182 *	−416 **	0.24 **	0.28 **	0.39 **	0.12	0.29 **	0.13	0.39 **	0.30 **	−0.02	−0.04

STR: *Striga*, DT: drought, OPT: optimum; *, ** significant at 0.05 and 0.01 probability levels, respectively; T1, T2, T3, and T4 = testers 1, 2, 3 and 4.

**Table 6:** Heterotic groups of 33 provitamin A inbred lines and four testers across contrasting environments in Nigeria, 2014–2016, using the HGCAMT grouping method.

Group 1	Group 2	Group 3
TZdEEI 7	TZEEIOR 30	TZdEEI 9
TZEEIOR 11	TZEEIOR 35	TZdEEI 13
TZdEEI 12	TZEEIOR 42	TZEEI 69
TZEEIOR 92	TZEEIOR 47	TZEEI 82
TZEEIOR 99	TZEEIOR 76	TZEEI 96
TZEEIOR 102	TZEEI 79	TZEEI 63
TZEEIOR 123	TZEEI 81	TZEEI 64
TZEEIOR 125	TZEEIOR 97	TZEEI 68
TZEEIOR 41	TZEEIOR 109	TZEEI 73
TZEEI 58	TZEEIOR 139	TZEEI 76
TZEEIOR 161	TZEEIOR 140	TZEEI 95
TZEEIOR 249	TZEEIOR 146	
TZEEIOR 251	TZEEIOR 197	

## Data Availability

The datasets used in the present study are available at the IITA CKAN repository.
